# Cells in experimental life sciences - challenges and solution to the rapid evolution of knowledge

**DOI:** 10.1186/s12859-017-1976-2

**Published:** 2017-12-21

**Authors:** Sirarat Sarntivijai, Alexander D. Diehl, Yongqun He

**Affiliations:** 1 European Molecular Biology Laboratory - European Bioinformatics Institute (EMBL-EBI), Wellcome Trust Genome Campus, Hinxton, Cambridge CB10 1SD UK; 2 Department of Biomedical Informatics, Jacobs School of Medicine and Biomedical Sciences, University at Buffalo, The State University of New York, Buffalo, New York 14203 USA; 3 Unit for Laboratory Animal Medicine, Department of Microbiology and Immunology, Center for Computational Medicine and Bioinformatics, and Comprehensive Cancer Center, University of Michigan Medical School, Ann Arbor, MI 48109 USA

**Keywords:** Cell culture, Cell line, Cell ontology, Cell line ontology, Cell types, human cell atlas

## Abstract

Cell cultures used in biomedical experiments come in the form of both sample biopsy primary cells, and maintainable immortalised cell lineages. The rise of bioinformatics and high-throughput technologies has led us to the requirement of ontology representation of cell types and cell lines. The Cell Ontology (CL) and Cell Line Ontology (CLO) have long been established as reference ontologies in the OBO framework. We have compiled a series of the challenges and the proposals of solutions in this CELLS (Cells in ExperimentaL Life Sciences) thematic series that cover the grounds of standing issues and the directions, which were discussed in the First International Workshop on CELLS at the the International Conference on Biomedical Ontology (ICBO). This workshop focused on the extension of the current CL and CLO to cover a wider set of biological questions and challenges needing semantic infrastructure for information modeling. We discussed data-driven use cases that leverage linkage of CL, CLO and other bio-ontologies. This is an established approach in data-driven ontologies such as the Experimental Factor Ontology (EFO), and the Ontology for Biomedical Investigation (OBI). The First International Workshop on CELLS at the International Conference on Biomedical Ontology has brought together experimental biologists and biomedical ontologists to discuss solutions to organizing and representing the rapidly evolving knowledge gained from experimental cells. The workshop has successfully identified the areas of challenge, and the gap in connecting the two domains of knowledge. The outcome of this workshop yielded practical implementation plans to filled in this gap.

This CELLS workshop also provided a venue for panel discussions of innovative solutions as well as challenges in the development and applications of biomedical ontologies to represent and analyze experimental cell data.

## Introduction

The rise of cell technologies is a double-edged sword. It has provided science with a fast lane to advance discovery in biomedical research. Experimental primary cell cultures and immortalized cell lines are widely used and often generated in a de novo fashion at the laboratory. Meanwhile, normalization of experimental cell data produced in different laboratory settings is sometimes difficult, even when the cell types studied are nominally the same. It has also become unclear where the separation between data and metadata is, due to the level of granularity and reproducibility of the details. Furthermore, there are no real unified modeling solutions that are universal to all experiments. Therefore, data representation and modeling is very much driven by individual experiments, causing inconsistency when a global consensus is needed. Consolidation of heterogeneous metadata in a large central data repository such as the Human Cell Atlas [1] is a major challenge. New knowledge obtained by high-resolution technologies such as CyTOF (mass cytometry) and single-cell RNA sequencing adds more data volume and metadata complexity, which require robust representation, especially regarding novel cell populations that do not belong to existing classes of CL or CLO. For these reasons, the community needs a clear distinction between data and metadata. At the ICBO-CELLS workshop, we discussed how ontologies support the modeling, representation, and analysis of cell-related data, metadata, and knowledge gained from experimental cell studies. After the discussion, we came to an agreement that both experimental and computational scientists in this domain would benefit from a shared consensus cell metadata model which can only be derived by community participation, discussion, and collaboration wherever possible. The outcome of this workshop was deemed relevant to the international audiences from both industry and academia. In this thematic CELLS issue, it is the CELLS organizers’ hope that the general audience, as well as seasoned experimentalists would find the discussion extremely useful to designing and implementing an experimental cell metadata framework in large and complex enterprises.

Cell cultures are a crucial component in life science experiments. Cells are versatile and can be used in many domains such as vaccine and drug development, developmental biology, and large-scale genomics studies. Even though there exist robust representations of cells and cell lines, there remain the issues of fast-evolving technologies that produce large amounts of data that require flexible ways of cell representation like never before seen. There remains room for improved outreach into the experimental research domain by ontologists as demonstrated in studies presented at this CELLS workshop.

The workshop covered two main areas: (i) the extension of CL and CLO for ontological representation of cell types and cell lines in new technologies and experiments, and (ii) applications and challenges in real-world use cases which may require other ontological adaptations beyond CL and CLO. Examples of biomedical subject matters in the scope of this workshop were: (i) the relationship of cell components to cell types and other biomedical entities and how that may impact health and disease biology (e.g. how the subcellular component composition of certain cancer cell types reflects the prognosis and affects the progression of that cancer), (ii) in vitro - in vivo relationships between primary cell samples and related immortal cell lines, and (iii) modeling of cell responses and fast-growing cell-related data generated by new technologies (e.g. novel cell types identified via interpretation of CyTOF and single cell RNA-Seq data).

### Challenges in semantic representation of cell biology

There has always been an invisible wall between the experimental biologists and the informaticians in the world of bioinformatics. We have operated and managed to cross this boundary from time to time by the requirements of each project. However, in the era of the technologies advancing ever so rapidly, both on the biomedical and computational sides, we suddenly find ourselves lost in translation. High-content single-cell technologies have exemplified a scenario where both bio- and -informatics must come together and merge at the intersection of knowledge. It requires a deep understanding on how the data are produced biologically and processed computationally to build a big picture that can present an integrative view of human body at large in a granular-detailed level. The Open Biological/Biomedical Ontologies (OBO) Foundry has been founded and functional for over a decade [2], but it is not uncommon to find that many of us on the experimental side remain unaware of many controlled vocabularies and ontologies established under the OBO’s umbrella. Tools and database infrastructures are being built to accommodate high-complexity biological knowledge such as scMap to cluster and classify cell types of single-cell information based on the expression level [3], or Stemformatics knowledgebase for stem cell derived cell lines [4]. Managing and processing data in such projects can benefit from existing structured controlled vocabularies such as those established in the biomedical ontology domain. Superimposing standard vocabularies not only allows the database management to logically link diverse experimental components together, but also allows integration of new knowledge into existing frameworks. Analysis of the cell nomenclature usage in literature also reveals that despite decades of research exploiting in vitro cells to investigate in vivo behavior, reconciliation of standard cell vocabularies remain a challenge as shown in the study by Kafkas et al. in this issue.

We have also observed that, even within the biomedical ontology community, advances in biomedical technology present a similar challenge of terminology coverage. Even though weathered ontologists are fully aware of existing standards, finding the right term for their annotation is still proving difficult. Keeping up with evolving knowledge is not only about defining new terms, but also ensuring that the creation of new terms is absolutely required as this may result in an inflation of terms if requests to create new ontology terms are not reviewed carefully. New knowledge requires new vocabularies, but when do we stop? Or where do we begin? When should new knowledge be qualified for a new term? It is not surprising that, despite the years of collective comprehensive understanding of ontologies, we are still asking the question of data or metadata, or in other words, database versus ontology. The complexity of cell biology may also contribute to this debate. When a cell can cleanly be classified with logical axioms such as those shown in the retinal bipolar cells by Osumi-Sutherland et al. (BMC Bioinformatics, this issue), there may exist another case where it may not be as straightforward to do so as shown by Bakken et al. in their attempt to perform high-content classification of brain cells (BMC Bioinformatics, this issue).

While we are aware that the unsettling sea of reference cell type classification of high-throughput data is out there, the quest to scientific discovery cannot wait. The research vessels do not stay ashore. There are applications that are trying their best to sail through this stormy sea by patching their ships to stay afloat while waiting for this storm to pass. The NIH Common Fund Library of Integrated Network-based Cellular Signatures (LINCS) program investigates the molecular and cellular activities and has defaulted their data annotation to the CLO illustrates this urge to move forward (LINCS knowledge representation, Ong. E et al., this issue). There are also attempts to ease the normalization of the diversified experimental cell standards such as shown by Ong et al. in their effort to align cell line information in the Experimental Factor Ontology (EFO) with the cell line classes in CLO (EFO-CLO alignment, Ong. E et al., this issue). It should also be noted here that questions of differentiating primary cell lines from immortalized cell lines has also doubly stressed the importance of identifying data and metadata and drawing a clear line between them as hinted in this study.

### Proposal of solutions by the community on the semantics of cell biology

It has become clear that the challenges that we are faced with today cannot be resolved by one entity alone. Neither the biologist community nor biomedical ontologist community alone can overcome this issue. This raises the level of urgency to the point never before seen that both communities need to come together in a timely manner. Fostering collaboration between both experimental and computational scientists requires a venue for discussions so that the discussions can turn themselves into a fruitful production - implementation and improvement of the semantic framework, and practical training on how the biologist users can fully exploit ontologies in their data annotation and analysis. This is a continuous process that will benefit from the feedback input to refine the solutions. To date, the CELLS workshop is the first attempt of its kind to establish the dialogue to move forward in finding the optimal solutions to these challenges. The workshop has laid out the communication channels between the Human Cell Atlas (HCA) ontologists with the Cell Ontology developers that connect back to the experimental biologists generating and analyzing single-cell data. The discussion has resulted in an agreement for the HCA ontologists to request modifications and extensions to the existing CL and CLO.

Furthermore, bringing the bench researchers who are experts in laboratory experiments to the first ICBO-CELLS workshop has exposed them to the ontology development and considerations, and at the same time, the ontologists have gained insight to the hands-on activities at the bench. This has stressed on the importance of outreach and bringing awareness of the different natures of interpretation of biology knowledge that inevitably exist on both sides of research which asks the same question. We have identified the area where the use of biomedical ontologies can aid effective data annotation for the scMap tool and Stemformatics database to extend the analysis/content of the resources to other domain knowledge beyond their defined project scopes. It is agreed that the outreach activity like the one exemplified at ICBO-CELLS workshop is very much needed and this should be promoted in both experimental biology and biomedical ontology communities where possible.

When investigating how to best represent the cell modeling with ontologies, two main solutions were proposed. Should we take the Rector normalization approach [5] in building design patterns for the classification of new cell types with granular details of cell attributes such as those exemplified in bipolar neuronal cells (Osumi-Sutherland, this issue)? Or should we extend the ontology by directly asserting the new classes with axiom declarations to supplement the details of the new class attributes when examining cells with detailed surface marker proteins? (Bekken et al., this issue) The two approaches may be deemed appropriate in different biological circumstances. Solutions are open to discussion, with an additional point to consider: can we adapt the two approaches so that they can be compatible with each other to suit the different scenarios where one of them is more suitable than the other based on the use case? The ontologists present at this workshop are engaged in active communication to find the most optimal solution to this question.

## Discussion

As shown in the few scenarios here, it is clear without a doubt that experimental cell standardized nomenclature is a very important driving mechanism to move high-content single-cell research forward. The challenge of knowledge representation is paramount, and it requires all parties, both biologists and ontologists to come together and try to find the common ground where everyone can mutually benefit. The CELLS workshop is the first of its kind to address this, and provide a venue for discussion where the proposal of solutions was drawn by all parties involved. Although many topics were discussed at the workshop, there remain other issues that need to be taken into consideration when dealing with the high complexity of single-cell biology. In addition to the requirement of using different approaches to data representation for different biological systems of the body, the question of capturing spatial information of the single cell in the scope of the tissue/organ is a critical aspect of understanding single-cell biology in the context of the whole organism. The Common Coordinate Framework meeting organized by the National Institute of Health, Chan-Zuckerberg Initiative, and the Human Cell Atlas as part of the Human Biomolecular Atlas Program (HuBMAP) [6] and the Human Cell Atlas initiative lays the foundation for a framework to systematically describe the coordinates of the human body with a semantically consistent set of vocabularies and structures. The HCA ontologists are working closely with the experimental biology experts to establish a computational metadata framework that can be linked back to the ontology-aided content and analyses seamlessly.

Accessibility and exposure to the existing ontologies and standards is another long-standing challenge that will continue to be one key aspect into building a seamless computational connectivity among the high-complexity biological components. One cannot stress enough the importance of transparency and outreach of the implementation inside the biomedical ontology world. Even though all the activities at the OBO Foundry have always been open, most OBO Foundry outreach and maintenance efforts are based on voluntary effort. Outreach has always been a challenge for OBO Foundry caretakers. However, with the increasing interest and necessity that drives data integration today, the awareness of the need to use existing standards has also increased. With more conversation centered around reusing standards and FAIR principles [7], channeling users to an open-access resource such as the OBO Foundry should gain more engagement from the community to promote the accessibility of the open (meta)data.

Modeling stem cell derived cell lines will continue to be a difficult challenge. The multiple possible end products resulting from the unique characteristics of in vitro stem cell cultures such as those seen in work on induced pluripotent stem cells introduce complexity to the knowledge modeling for stem cells. Probabilities cannot be asserted directly into the ontology, but rather using other modeling techniques such as nanopublications [8] or OBAN [9] to avoid logical errors when reasoning with the ontology. Ontology design patterns utilizing OWL semantics, complemented by the probability modeling techniques, though harder, will be an optimal solution to resolving the non-static modeling of stem cell derived cell lines.

It is our hope that, while we are developing the solutions to overcome the challenges described in this CELLS issue, other scientific quests for knowledge discovery will do their best to get through this time of dynamic knowledge evolution. Through engaging users who are neither the standards/ontology developers nor the experimental biologists, but rather the middle-man consumers of data and implementation (such as the database maintenance staff, or the bioinformaticians trying to model rapidly-changing biological content), they will be informed of current efforts in finding the solutions. This will allow them to design their systems to be flexible in handling upcoming changes resulting from knowledge discovery, and plan ahead to allow their implementation to support such changes. Dialogue across the different communities as facilitated by the CELLS workshop will lay a foundation of communication and outreach. This will not be a one-time off that can resolve all difficulties, but rather an ongoing effort that will continue for the next years to come.
